# The roles of *SAP2*, *STP1*, and *MRR2* on biofilm formation and itraconazole resistance through autophagy in *Candida albicans*

**DOI:** 10.3389/fmed.2026.1817039

**Published:** 2026-05-21

**Authors:** Jing Yang, Wenli Feng, Mingjun Xie, Daiyao Yu, Wenqin Yang, Yan Ma, Zhiqin Xi

**Affiliations:** Department of Dermatovenereology, The Second Hospital, Shanxi Medical University, Taiyuan, Shanxi, China

**Keywords:** autophagy, biofilm, *Candida albicans*, itraconazole resistance, MRR2, Sap2, STP1

## Abstract

**Background:**

*Candida albicans* biofilm formation and azole antifungal resistance are major obstacles to clinical treatment of candidiasis. *STP1* and *MRR2* are implicated in *C. albicans* virulence and stress responses, while *SAP2* plays critical roles in biofilm formation and drug resistance. However, the regulatory mechanisms of *STP1* and *MRR2* on *SAP2* expression, biofilm formation, and itraconazole (ITR) resistance, particularly via autophagy, remain unclear.

**Methods:**

We compared biofilm-forming ability (via crystal violet assay and microscopy), ITR susceptibility, and autophagic responses among standard ATCC11006, *STP1*∆/∆, *MRR2*∆/∆, ITR-sensitive, and ITR-resistant strains. Gene expression levels of *SAP2*, and key autophagy-related genes were quantified using RT-qPCR under planktonic and biofilm conditions. The role of autophagy was further investigated using the inducer rapamycin and nitrogen starvation assays.

**Results:**

ITR-resistant strains exhibited significantly stronger biofilm-forming ability than sensitive strains. *STP1* deletion enhanced biofilm formation, while *MRR2* deletion impaired it. *SAP2*, *STP1*, and *MRR2* expression was significantly higher in resistant strains than in sensitive strains under both planktonic and biofilm states, with a significant positive correlation between *SAP2* and *MRR2* under planktonic conditions (*r* = 0.659, *p* = 0.002). Notably, expression of all tested autophagy-related genes was significantly upregulated in biofilms and in ITR-resistant strains, but was markedly downregulated in both *STP1Δ/Δ* and *MRR2Δ/Δ* strains. Rapamycin induced autophagosome formation and upregulated *SAP2* and *ATG* gene expression, but this response was blunted in the knockout strains. Furthermore, both *STP1Δ/Δ* and *MRR2Δ/Δ* strains displayed increased sensitivity to nitrogen starvation.

**Conclusion:**

Our findings demonstrate that *STP1* and *MRR2* are critical regulators of biofilm formation and ITR resistance in *Candida albicans*. They exert these effects, at least in part, by modulating the expression of *SAP2* and, importantly, by governing the autophagic pathway.

## 1 Introduction

*Candida albicans* is an opportunistic fungal pathogen that normally exists as a commensal microorganism in humans, colonizing various mucosal surfaces such as the oral cavity, gastrointestinal tract, and genital tract without causing symptoms (1). However, when the host’s barrier function or immune system is impaired, for instance, in cases of HIV infection, long-term use of antibiotics or immunosuppressants, and diabetes mellitus, *C. albicans* can switch from a commensal to a pathogenic state, triggering a spectrum of infections ranging from superficial mucosal candidiasis to life-threatening invasive candidiasis (IC) (2, 3). Clinically, IC is associated with a high mortality rate (30–50%) among critically ill patients, posing a significant challenge to modern healthcare systems (4). As the second most common fungal pathogen globally and the fourth leading cause of bloodstream infections, *C. albicans* imposes a substantial global economic burden and public health threat (5). Despite the widespread use of antifungal agents, over 250,000 deaths annually worldwide are attributed to invasive candidiasis and candidemia (6). Currently, clinical treatment of *C. albicans* infections relies primarily on three major classes of antifungal drugs: azoles, polyenes, and echinocandins. Among these, azoles, including fluconazole (FCA), itraconazole (ITR), and voriconazole (VRC), are the most widely used due to their broad spectrum of activity and favorable pharmacokinetic profiles (7). These agents exert their antifungal effects by inhibiting the activity of cytochrome P-450-dependent enzyme lanosterol 14α-demethylase (CYP51), thereby blocking the biosynthesis of ergosterol, a key component of the fungal cell membrane (8, 9). However, the escalating incidence of azole resistance in *C. albicans*, driven by the inappropriate long-term use of antifungal drugs and the pathogen’s inherent adaptive capacity, has become a pressing concern (10). Thus, exploring the pathogenic and drug-resistant mechanisms of *C. albicans*, identifying novel antifungal targets, and developing next-generation antifungal agents are urgently needed.

The pathogenicity of *C. albicans* is attributed to multiple virulence factors, with biofilm formation recognized as a pivotal determinant (11). Biofilms are complex, matrix-encased microbial communities that adhere to biotic or abiotic surfaces, endowing *C. albicans* with enhanced resistance to host immune defenses and antimicrobial agents (12, 13). The process of *C. albicans* biofilm formation typically involves four stages: adhesion, proliferation, maturation, and dispersion. Current research on biofilm-associated drug resistance has focused on three main mechanisms: overexpression of efflux pump-encoding genes, the physical barrier of the extracellular matrix, and the formation of dormant persister cells (14, 15). Clinical studies have demonstrated a significant correlation between *C. albicans* biofilm-forming ability and mortality in patients with candidemia, with biofilm-forming isolates exhibiting markedly increased drug resistance (16, 17). The biofilm matrix, composed of extracellular polysaccharides, proteins, and nucleic acids, acts as a physical barrier that restricts drug penetration, while altered metabolic activity and persister cell formation within the biofilm further contribute to antifungal tolerance (18, 19). Therefore, understanding the molecular mechanisms underlying *C. albicans* biofilm formation is crucial for developing novel therapeutic strategies to combat recalcitrant candidiasis.

Autophagy, an evolutionarily conserved intracellular degradation and recycling process, is implicated in fungal growth under drug-induced stress and nutrient limitation This process involves the sequestration and degradation of damaged organelles or cytoplasmic proteins to meet cellular metabolic demands, facilitate organelle renewal, and promote self-repair (20). Moderate autophagy is beneficial for cellular growth, whereas excessive autophagy may induce cellular damage. Recent studies have confirmed the presence of autophagy in fungal yeasts, including *C. albicans*, and have linked this process to drug resistance (21, 22). Yeast autophagy can be activated through multiple pathways, such as rapamycin (a classic autophagy inducer), nutrient deprivation, and reactive oxygen species (ROS) accumulation (23). In *C. albicans* physiology, autophagy plays a dual role: it promotes cell survival under nutrient starvation and environmental stress, while also contributing to biofilm development and antifungal tolerance (24, 25). However, the precise regulatory networks connecting autophagy to biofilm formation and ITR resistance remain incompletely elucidated.

Secreted aspartic proteases (Saps) are key virulence factors of *C. albicans*. They not only catalyze the hydrolysis of host cell membrane proteins to enhance adhesion and tissue invasion but also neutralize cellular and molecular components of the host defense system, enabling *C. albicans* to evade or resist antimicrobial attacks (26). Among the Sap family, Sap2 is a critical pathogenic factor that plays pivotal roles in *C. albicans* biofilm formation, virulence, and drug resistance (27). Overexpression of *SAP2* enhances the ability of *C. albicans* to adhere to and invade host tissues, promotes biofilm maturation and structural stability, and confers increased invasiveness and drug resistance, ultimately elevating the minimum inhibitory concentration (MIC) of antifungal agents and predisposing to fatal candidemia (28, 29). Furthermore, the aberrant expression of *SAP2* is regulated by multiple transcription factors, including *CAP1*, *GCN4*, *MRR1*, *MRR2*, and *STP1* (30–32). However, the mechanisms by which key transcription factors such as *STP1* and *MRR2* regulate *SAP2* overexpression, thereby influencing biofilm formation and azole resistance, remain poorly understood.

*STP1* and *MRR2* are two genes implicated in *C. albicans* virulence and stress responses. *STP1*, a transcription factor belonging to the zinc finger family, regulates nitrogen metabolism, including the SPS amino acid-sensing pathway and the rapamycin-binding protein TOR pathway (33). Recent studies have shown that activated *STP1* upregulates genes involved in extracellular protein degradation and peptide uptake, including *SAP2*, enabling fungal pathogenesis in the host; conversely, *STP1*-deficient cells fail to express *SAP2* (33, 34). Additionally, both *STP1* and *SAP2* are subject to nitrogen catabolite repression (NCR), with NCR-mediated regulation of *SAP2* occurring via modulation of *STP1* expression, ultimately enhancing *C. albicans* virulence (35). Emerging evidence suggests that *STP1*-induced autophagy under nitrogen induction promotes *SAP2* overexpression, influences biofilm formation, and contributes to host pathogenesis (36). *MRR2* encodes a putative magnesium transporter that also plays a role in biofilm formation. By constructing *MRR2* mutants, researchers have demonstrated that *Mrr2p* is involved in the adhesion and colonization of *C. albicans* during biofilm formation in a murine kidney model (37). Another study investigated the role of *MRR2* in *C. albicans* drug resistance using *MRR2*∆/∆ strains, revealing that *MRR2* could modulate resistance by regulating the expression of the multidrug efflux pump gene *CDR1* (38). Our previous study has further confirmed that *MRR2* overexpression promotes *CDR1* upregulation, mediating FCA resistance and enhancing *C. albicans* virulence (39). However, the roles of *STP1* and *MRR2* in biofilm formation and ITR resistance, particularly through the regulation of autophagy, have not been systematically investigated.

Given the clinical significance of *C. albicans* biofilm-associated infections and the rising prevalence of azole resistance, the present study aimed to explore the interactions between *STP1*/*MRR2* and *SAP2*, as well as their roles in biofilm formation and ITR resistance. Therefore, we hypothesized that *STP1* and *MRR2* may regulate *SAP2* overexpression, thereby influencing *C. albicans* biofilm formation and further enhancing virulence and drug resistance via autophagy. To validate this hypothesis, we compared biofilm-forming ability, ITR susceptibility, and autophagic responses among reference *C. albicans*, *STP1*-deficient, and *MRR2*-deficient strains. Additionally, we analyzed the expression of autophagy-related genes and virulence factors to preliminarily elucidate the underlying molecular mechanisms. The findings of this study will provide new insights into the regulatory networks governing *C. albicans* biofilm formation and drug resistance, potentially identifying novel targets for antifungal drug development.

## 2 Materials and methods

### 2.1 Experimental strains

The reference strain of *C. albicans* ATCC11006 was purchased from the Fungus and Mycosi Research Center, Peking University Medical Science (Beijing, China). The 10 ITR-resistant strains (17r, 40r, 54r, 58r, 67r, 68r, 96r, 105r, 106r, and 188r), and 10 FCA, ITR or VRC-sensitive strains (3 s, 4 s, 26 s, 34 s, 78 s, 95 s, 150 s, 157 s, 174 s, and 176 s for *STP1*-related experiments; as well as 78 s, 81 s, 87 s, 94 s, 95 s, 117 s, 123 s, 150 s, 156 s, and 194 s for *MRR2*-related experiments) were identified and provided by the Dermatovenereal Fungus Laboratory of The Second Hospital of Shanxi Medical University (Shanxi, China). Additionally, the *C. albicans STP1* gene-deficient strain (*STP1*∆/∆) and the *C. albicans MRR2* gene-deficient strain (*MRR2*∆/∆) were homozygous full-gene double-allele knockout mutants derived from the Noble & Johnson genome-wide deletion library, with the genetic background of *C. albicans* SN152. These mutant strains do not require amino acid supplementation for growth under standard YPD culture conditions, as well as were kindly provided by Professor Changbin Chen at Shanghai Pasteur Institute, Chinese Academy of Sciences (Shanghai, China).

### 2.2 Preparation of *Candida albicans* under planktonic and biofilm conditions

*C. albicans* was inoculated on YPD Agar Medium (containing 10 g/L of peptone, 5 g/L of yeast extract, 10 g/L of glucose and 10 g/L of agar; Beijing Aoboxing Biotechnology Co., Ltd., Beijing, China) and incubated at 37 °C for 72 h to ensure full recovery, stabilize consistent colony morphology, and achieve uniform physiological status after two successive subculturing steps, not because *C. albicans* requires 72 h to achieve visible growth. After two successive subculturing steps, single colonies were inoculated into 5 mL of YPD liquid medium (containing 10 g/L of peptone, 5 g/L of yeast extract, and 10 g/L of glucose, Beijing Aoboxing Biotechnology Co., Ltd), and cultured at 30 °C with orbital shaking (220 rpm) for 24 h. Then, 1 mL cultured fungal solution was centrifuged at 50000 rpm for 2 min, and the supernatant was discarded. The sediments were washed with PBS three times, and resuspended in YPD liquid medium to obtain the *C. albicans* under the planktonic condition.

For biofilm formation, the aforementioned *C. albicans* suspension (100 μL with a density of 5 × 10^6^ CFU/mL) was added to each well containing 100 μL of YPD liquid medium of a 96-well plate. After incubation at 37 °C for 1 h, each well was gently washed twice with sterile PBS to remove non-adherent (planktonic) cells. Then, 100 μL of fresh YPD liquid medium was added to each well, and the plate was incubated at 37 °C for a further 12 h. After incubation, each well was gently washed three times with sterile PBS, and100 μL of fresh YPD liquid medium was added again, followed by incubation at 37 °C for an additional 12 h. Finally, the wells were gently washed three times with sterile PBS, the medium was completely removed, and the plate was incubated at 37 °C for an additional 24 h to allow maturation of the biofilm.

### 2.3 Determination of *in vitro* biofilm formation ability

*In vitro* biofilm formation ability was assessed using Calcofluor White Stain and crystal violet staining. Calcofluor White Stain was used to observe the detailed morphology and structural characteristics of the cellular and hyphal elements within the biofilms using a confocal laser scanning microscopy. Crystal violet staining was used for inverted microscopy observation of biofilm dynamics and quantitative measurement of total biofilm biomass at 48 h (OD_490nm_).

For Calcofluor White Stain, biofilms at 1 h, 12 h, 24 h, and 48 h were fixed with methanol for 20 min, and added with Calcofluor White Stain. After cultured in the dark at 37 °C for 30 min, the biofilms were washed with PBS, and visualized using a confocal laser scanning microscopy (Leica Microsystems, Vizna, Germany) at a magnification of 200 ×.

For crystal violet staining, the fixed biofilms at 1 h, 12 h, 24 h, and 48 h were stained with 1% crystal violet (100 μL) at 37 °C for 30 min, and then washed with PBS. Then, 100 μL of 33% glacial acetic acid was added for 2 min, and the absorbance at 490 nm was measured using a microplate reader. Or after air dry, the biofilms were observed under an inverted microscope at a magnification of 200 ×.

### 2.4 Spot assay of *Candida albicans*

For preparation of rapamycin culture medium, 400 mL double-distilled water, 10 g of peptone, 10 g of glucose, 10 g of agar, 5 g of yeast extract were used. After sterilization, diluted rapamycin solution was added to achieve the final concentrations of 15 nM and 20 nM in the YPD solid medium, respectively. Prior to solidification, the medium was poured into sterile disposable Petri dishes and stored at 4 °C until further use. For preparation of nitrogen-deficient culture medium, 400 mL double-distilled water, 3.35 g of yeast nitrogen base (YNB), 10 g of glucose, and 15 g of agar (added exclusively for preparing solid medium) were added, and after complete dissolution, the final volume of the solution was adjusted to 500 mL. After sterilization, the liquid medium (without agar supplementation, SD-N) was directly aliquoted and stored at 4 °C until further use. For the solid medium, 0.04 mg/mL of L-arginine hydrochloride (SD-N + Arg) and 0.025 mg/mL of L-leucine (SD-N + Leu) were incorporated aseptically while the medium remained in a molten state (prior to solidification). The supplemented solid medium was then poured into sterile disposable Petri dishes. After complete solidification, the Petri dishes were sealed hermetically and stored at 4 °C for subsequent experimental applications.

In addition, for spot assay, a 1 mL of the fungal suspension, which had been cultured for 16 h, was transferred into an Eppendorf (EP) tube. After centrifugation, the resulting pellet was rinsed thoroughly with sterile PBS for three consecutive times. The washed pellet was then resuspended in PBS to adjust the final concentration of the fungal suspension to 5 × 10^6^ CFU/mL. After that, a 10 μL of the fungal suspension was spotted sequentially onto the prepared solid agar plates (such as YPD medium, YPD medium containing 15 nM and 20 nM rapamycin, SD-N, SD-N + Arg, or SD-N + Leu). The plates were air-dried under sterile conditions and then incubated upside down at 37 °C in a constant-temperature incubator. Fungal growth was monitored and recorded at predetermined time intervals.

### 2.5 Measurement of fungal tolerance to nitrogen starvation

A single colony was picked from the solid agar plate and inoculated into 5 mL of YPD liquid medium. The inoculated medium was incubated at 30 °C with shaking at 200 rpm in a constant-temperature shaker for 16 h. Then, a 1 mL of the fungal suspension was transferred into an EP tube, and washed three times with PBS. The washed fungal pellet was resuspended separately in YPD medium and nitrogen-deficient medium, respectively, to adjust the fungal concentration to 1 × 10^6^ CFU/mL with a final volume of 5 mL for each group. After incubated at 30 °C with shaking at 200 rpm in a constant-temperature shaker, the viable fungal count was determined every 24 h, and each experiment was performed in triplicate to ensure reproducibility.

For viable count analysis, a 100 μL of the fungal suspension was uniformly spread onto YPD solid agar plates. The plates were air-dried under sterile conditions and then incubated at 37 °C in a constant-temperature incubator. After 24 h of incubation, the number of single colonies on each solid agar plate was counted. Based on the colony count results, a fungal relative growth rate curve was plotted.

### 2.6 Observation of autophagosomes by a laser scanning confocal microscope under differential interference contrast (DIC) mode

The experiment was divided into the control and treatment groups. For the control group, the standard strain ATCC11006, *MRR2*∆/∆ strain, sensitive and resistant strains were inoculated into 5 mL of YPD liquid medium separately, and incubated at 30 °C with shaking at 200 rpm for 16 h, after which the fungal cells were resuspended in fresh YPD liquid medium. Meanwhile, phenylmethylsulfonyl fluoride (PMSF) was added to achieve a final concentration of 1.5 mM, followed by further incubation at 30 °C for 16 h. For the treatment group, the standard strain ATCC11006 and *MRR2*∆/∆ strain were first cultured by incubation at 30 °C with shaking at 200 rpm for 16 h. A 1 mL of the cultured fungal suspension was then added with rapamycin to reach a final concentration of 200 nM. Simultaneously, PMSF was added to obtain a final concentration of 1.5 mM, and the mixture was induced for 7 h. After incubation, both the control group and the treatment group were washed with PBS to remove the culture medium. A 5 μL of each fungal suspension was spotted onto glass slides pre-soaked in polylysine solution, and then the slides were observed under a laser scanning confocal microscope in DIC mode.

### 2.7 RNA extraction and real-time quantification PCR (RT-qPCR)

A single colony of *C. albicans* was picked from the YPD solid agar plate and inoculated into 5 mL of YPD liquid medium. The culture was incubated overnight at 30 °C with shaking at 200 rpm in a water bath shaker. For biofilm samples, mature biofilms were washed with PBS, scraped with RNase-free cell scrapers, and collected for RNA extraction. RNA extraction, reverse transcription, qPCR, and data analysis were performed identically for planktonic and biofilm samples.

Total RNA of *C. albicans* was extracted strictly following the manufacturer’s instructions of the column-based yeast total RNA extraction and purification kit (Sangon Biotech, Shanghai, China). Thereafter, the extracted RNA was subjected to reverse transcription, and the reaction system contained 15 μL RNA, 4 μL MonScript™ 5xRTIII All-in-One Mix, and 1 μL MonScript™dsDNase. The thermal cycling program was set up under the following conditions: incubation at 37 °C for 2 min, followed by incubation at 55 °C for 15 min, and final incubation at 85 °C for 5 min. Upon completion of the reaction, the resulting cDNA was retrieved and used for subsequent RT-qPCR analysis. The sequences of all primers were shown in Table 1; as well as *ACT1* was used as a housekeeping gene. The RT-qPCR reaction was initiated at 95 °C for 3 min and 95 °C for 5 s, followed by 40 cycles of 60 °C for 20 s and 95 °C for 15 s, as well as melting curves of 60 °C for 60 s, 95 °C for 15 s, and 60 °C for 15 s. The standard strain ATCC11006 was designated as the control group. The relative quantification of target gene expression was calculated using the 2^-ΔΔCt^ method, where the ΔΔCt = (Ct value of the target gene – Ct of the reference gene) _experimental group_ − (Ct value of the target gene – Ct value of the reference gene) _control group._ The value of 2^−ΔΔCt^ was defined as the relative expression level of the target genes.

**Table 1:** Sequences of all primers.

Primer	Sequences (5′-3′)	Length
*ACT1*	F: TAGGTTTGGAAGCTGCTGGT R: ACGTTCAGCAATACCTGGCA	137 bp
*SAP2*	F: CCAATGAAGCCGGTGGTAGT R: TATTTGTCCCGTGGCAGCAT	119 bp
*SPT1*	F: CCACCGCCATTCCACCAACAC R: AGACTCGACATCATCTGAGGAGACC	111 bp
*MRR2*	F: GCCTCAACCCGAACTTTAGATTTGC R: GGTAGATGTGCTGGAAGAAGGTGTC	123 bp
*TOR*1	F: TTGGCAATCAGATGGCGACT R: GTCTCCTTTCTGCTCGCTGT	190 bp
*ATG1*	F: GGCAAGAAACGAGCGAATCC R: GACAGCTCTCGTCTGCACTT	187 bp
*ATG2*	F: AGTAGCATTCCTGTTGGCCC R: CTTGTTCGCCTTTTCGGTGG	171 bp
*ATG5*	F: CCATGACTTGGGTTGCTGGA R: TCAATTTGTCCATCCCCCGC	105 bp
*ATG7*	F: TGAGGCATGGAGTGACGAAC R: CCATCAATGCACCTCCTGGT	138 bp
*ATG8*	F: AGTGCCAGTGGATTTGACTGT R: AGCGGTTGGGGGTAATATGTC	115 bp
*ATG11*	F: CAACGGCACTCCAAGCTCTA R: TCACTCTCGGTTCCAGACGA	120 bp
*ATG12*	F: TCGGTTCAACACCGTCGATT R: CGAGCCTATCTGCTCGTCAG	164 bp

### 2.8 Statistical analysis

Statistical analyses were performed using SPSS 27.0 software, and graphs were generated with GraphPad Prism 9.0 software. For data that conformed to a normal distribution, Student’s t-test was conducted, and the results were expressed as mean ± standard deviation (SD). For data that did not follow a normal distribution, the Kruskal-Wallis H test (rank sum test) was applied, and the results were presented as median ± interquartile range (M ± IQR). Correlation analyses were separately performed between *SAP2* and *STP1*, as well as between *SAP2* and *MRR2*. A *p*-value < 0.05 was considered statistically significant.

## 3 Results

### 3.1 Biofilm formation in different *Candida albicans* strains

**Figure 1:**
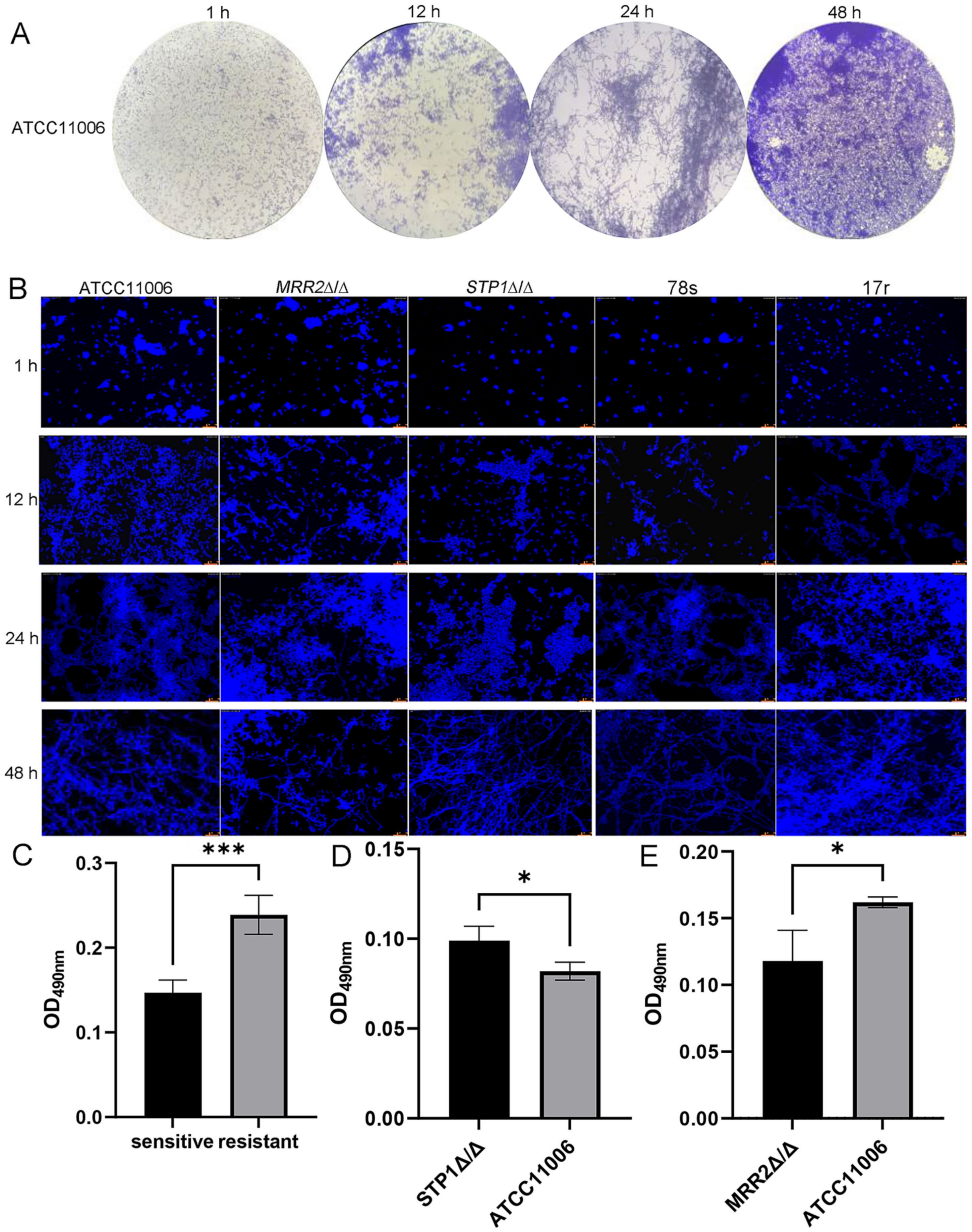
Biofilm formation in different *Candida albicans* strains. **(A)** Morphological structure of standard *Candida albicans* ATCC11006 biofilm *in vitro* observed under inverted microscope at 200 × magnification. **(B)** Biofilm morphology of *Candida albicans* (ATCC11006, *STP*1Δ/Δ, *MRR2*Δ/Δ, sensitive strain and resistant strain) observed *in vitro* under confocal laser scanning microscopy at 200 × magnification. Scale bar = 25 μm. **(C)** Comparison of the biofilm formation ability between the sensitive and ITR-resistant strains. **(D)** Comparison of the biofilm formation ability between ATCC11006 and *STP*1Δ/Δ strains. **(E)** Comparison of the biofilm formation ability between ATCC11006 and *MRR2*Δ/Δ strains. * *p* < 0.05, *** *p* < 0.0001.

Crystal violet staining of the standard strain (ATCC11006) revealed distinct morphological changes during biofilm development over time: at 1 h of incubation, a large number of budding yeast cells were observed, with no typical pseudohyphae present at this early; at 12 h, a small amount of hyphae had formed, with partial aggregation of *C. albicans* cells and hyphae, presenting the initial morphology of biofilms; at 24 h, the length of *C. albicans* hyphae was significantly increased, and the hyphae and cells aggregated markedly to form a reticular structure; as well as at 48 h, *C. albicans* hyphae and cells had spread across the entire visual field, forming a dense reticular biofilm (Figure 1A).

Calcofluor White stain specifically binds to chitin in the fungal cell wall and was used to visualize cell wall and hyphal structures of *C. albicans*, emitting blue fluorescence under fluorescence microscopy. In this study, strain 78 s was used as the representative sensitive clinical strain, and strain 17r was used as the representative ITR-resistant clinical strain. Laser scanning confocal microscopy observations revealed dynamic biofilm development across different incubation periods. At 1 h of incubation, a large number of yeast cells were distributed sparsely with no obvious hyphae observed, corresponding to the fungal adhesion and colonization stage. At 12 h, weak blue fluorescence was detected around the yeast cells, and a small amount of hyphae aggregated surrounding the yeast cells to form scattered microcolonies, which gradually fused over time. At 24 h, extensive yeast cells and hyphae aggregated into clusters and intertwined to form a reticular structure. The blue fluorescence intensity was significantly enhanced, accompanied by increased secretion of chitin that gradually covered the surface of microcolonies, indicating the formation of early-stage biofilms. At 48 h, the biofilm was dominated by the hyphal phase, with intense blue fluorescence observed throughout the visual field, forming large, dense-structured mature biofilms. Furthermore, comparative observations showed that the hyphae of ITR-resistant strains (such as 17r) were more densely packed than those of ITR-sensitive strains (such as 78 s) at 48 h of incubation. Meanwhile, compared with the standard strain ATCC11006, the *STP*1Δ/Δ and *MRR2*Δ/Δ strains exhibited higher degrees of hyphal aggregation (Figure 1B).

Subsequently, we determined the OD_490nm_ values of different *C. albicans* strains after crystal violet staining to assess the biofilm formation. Compared with the sensitive strains, the OD_490nm_ value in the resistant strains were significantly increased (*p* < 0.0001), indicating the ability of ITR-resistant strains to form biofilms *in vitro* was significantly higher than that of sensitive strains (Figure 1C). In addition, in comparison with the standard strain (ATCC11006), the OD_490nm_ value was significantly higher in the *STP*1Δ/Δ strain (*p* < 0.05, Figure 1D), while was evidently lower in the *MRR2*Δ/Δ strain (*p* < 0.05, Figure 1E). These implied that the ability to form biofilm was significantly increased in *C. albicans* with STP1 knockdown, whereas significantly decreased in MRR1 silencing *C. albicans*.

### 3.2 *SAP2*, *MRR2*, *STP1* expression in ITR-resistance/sensitivity strains, and correlations between *STP1*/*MRR2* and *SAP2* under different states

The mRNA expression levels of *SAP2*, *MRR2* and *STP1* in ITR-resistant and sensitive clinical strains under planktonic and biofilm states were detected by RT-qPCR, with statistical analysis shown in Table 2. For normally distributed data, the expression level was presented as mean ± SD, and the Kruskal-Wallis H test was used for non-normally distributed data (median ± interquartile range, M ± IQR). At the planktonic state, the *SAP2* expression in resistant strains (1.302 ± 0.388) was significantly higher than that in sensitive strains (0.798 ± 0.484) (*p* = 0.02); and *STP1* expression in resistant strains (1.771 ± 1.081) was also markedly elevated compared with sensitive strains (0.944 ± 0.218) (*p* = 0.008); while *MRR2* expression showed non-normal distribution, with the resistant strain (0.968 ± 0.223) significantly higher than the sensitive strain (0.364 ± 0.437) (*p* = 0.008, Table 2). At the biofilm state, the expression of all three genes was significantly upregulated in both resistant and sensitive strains compared with the planktonic state, and the expression in resistant strains was still significantly higher than that in sensitive strains (Table 2).

**Table 2:** *SAP2*, *STP1*, and *MRR2* mRNA expression levels in sensitive and resistant fungal strains, and their correlations under different states.

Gene	Group	State	*N*	Mean ± SD	*t*	M ± IQR	z	*P*
*SAP2*	Planktonic	Sensitive	10	0.798 ± 0.484	2.567	/	/	0.02
		Resistant	10	1.302 ± 0.388		/		
	Biofilm	Sensitive	10	2.585 ± 0.928	2.708	/	/	0.017
		Resistant	10	4.288 ± 1.760		/		
*MRR2*	Planktonic	Sensitive	10	/	/	0.364 ± 0.437	−2.647	0.008
		Resistant	10	/		0.968 ± 0.223		
	Biofilm	Sensitive	10	0.989 ± 0.401	/	/	2.869	0.01
		Resistant	10	1.535 ± 0.449	/	/		
*STP1*	Planktonic	Sensitive	10	0.944 ± 0.218	2.958	/	/	0.008
		Resistant	10	1.771 ± 1.081		/		
	Biofilm	Sensitive	10	2.703 ± 1.425	2.708	/	/	0.017
		Resistant	10	4.647 ± 1.512		/		

Correlation			
State	Gene	*r*	*P*
Planktonic	*SAP2*	0.659	0.002
	*MRR2*		
Biofilm	*SAP2*	0.363	0.115
	*MRR2*		
Planktonic	*SAP2*	0.217	0.359
	*STP1*		
Biofilm	*SAP2*	0.437	0.054
	*STP1*		

SD, standard deviation; M ± IQR, median ± interquartile range; r, correlation coefficient.

Further correlation analysis further revealed the regulatory relationship between *STP1*/*MRR2* and *SAP2* under different states (Table 2). At the planktonic state, there was a significant positive correlation between *SAP2* and *MRR2* expression (*r* = 0.659, *p* = 0.002), while no significant correlation was observed between *SAP2* and *STP1* (*r* = 0.217, *p* = 0.359). At the biofilm state, no significant correlations were found between *SAP2* and *MRR2* (*r* = 0.363, *p* = 0.115) or between *SAP2* and *STP1* (*r* = 0.437, *p* = 0.054), though the latter showed a marginal positive correlation trend.

### 3.3 Comparison of autophagy-related genes between different *Candida albicans* strains under different states

**Figure 2:**
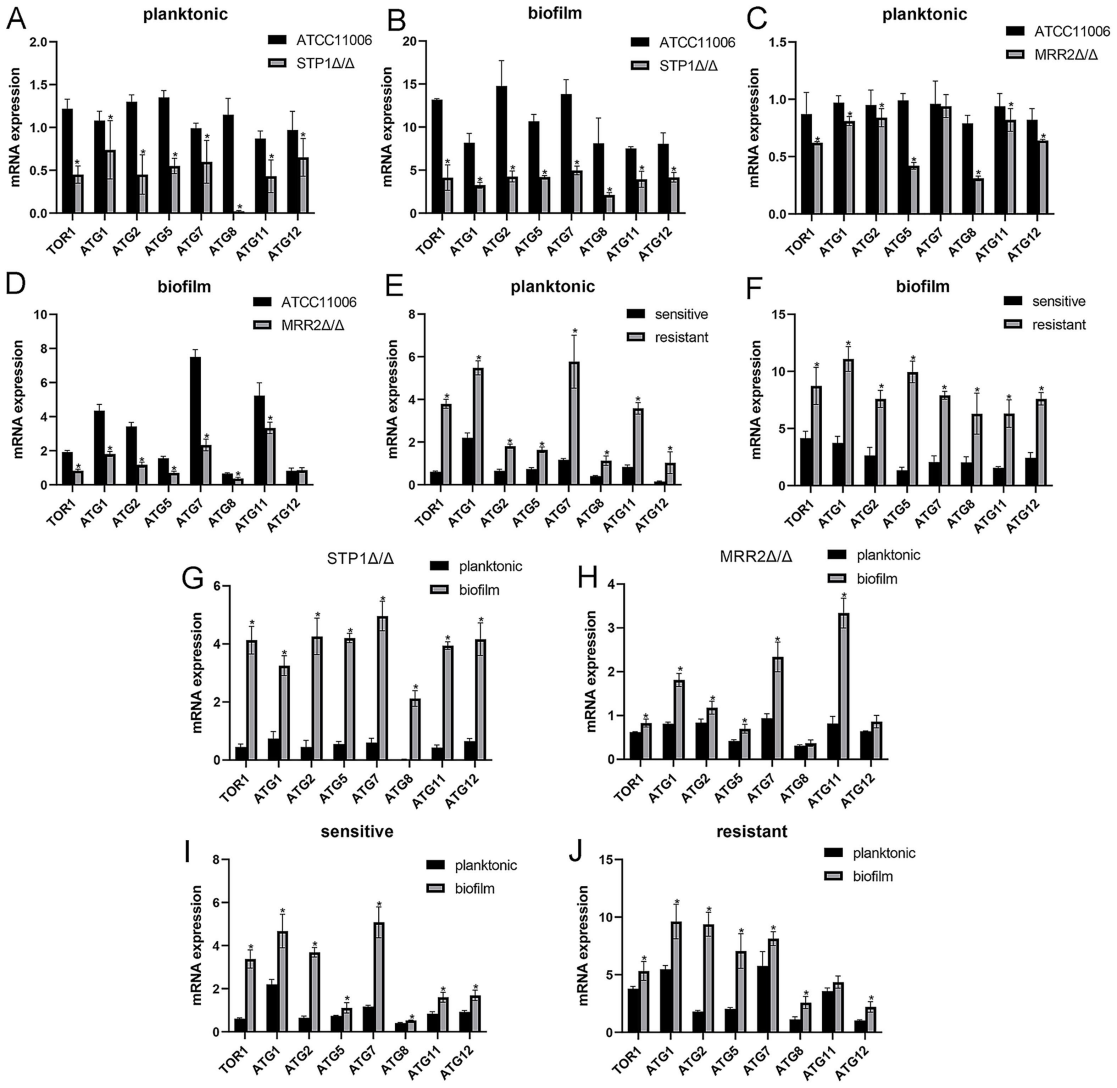
Comparison of autophagy-related genes between different *Candida albicans* strains under different states. The mRNA expression of autophagy-related genes between ATCC11006 and *STP*1Δ/Δ strains under planktonic **(A)** and biofilm **(B)** conditions. The mRNA expression of autophagy-related genes between ATCC11006 and *MRR2*Δ/Δ strains under planktonic **(C)** and biofilm **(D)** conditions. The mRNA expression of autophagy-related genes between the sensitive and resistant strains under planktonic **(E)** and biofilm **(F)** conditions. The mRNA expression of autophagy-related genes in *STP*1Δ/Δ **(G)** and *MRR2*Δ/Δ **(H)** strains between the planktonic and biofilm conditions. The mRNA expression of autophagy-related genes in sensitive **(I)** and resistant **(J)** strains between the planktonic and biofilm conditions. * *p* < 0.05.

To explore the involvement of autophagy in biofilm formation and ITR resistance, the mRNA expression of key autophagy-related genes (*ATG1*, *ATG2*, *ATG5*, *ATG7*, *ATG8*, *ATG11*, *ATG12*, and *TOR*1) was detected in different strains under planktonic and biofilm conditions (Figure 2).

Compared with the standard ATCC11006 strain, the *STP1*Δ/Δ strain showed significantly downregulated expression of all detected autophagy-related genes under both planktonic (Figure 2A) and biofilm (Figure 2B) states (*p* < 0.05). For the *MRR2*Δ/Δ strain, the expression of most autophagy-related genes was also significantly lower than that of the ATCC11006 strain under both planktonic (Figure 2C) and biofilm (Figure 2D) states (*p* < 0.05). Furthermore, both under planktonic (Figure 2E) and biofilm (Figure 2F) conditions, the expression of all detected autophagy-related genes in ITR-resistant strains was significantly higher than that in sensitive strains (*p* < 0.05). For the *STP1*Δ/Δ (Figure 2G) and *MRR2*Δ/Δ (Figure 2H) knockout strains, the expression of all detected autophagy-related genes at the biofilm state was significantly increased compared to that at the planktonic state (*p* < 0.05). A similar expression pattern was observed in clinical strains of both ITR-sensitive (Figure 2I) and resistant (Figure 2J) strains had significantly upregulated autophagy-related gene expression in the biofilm state compared with the planktonic state (*p* < 0.05).

### 3.4 Effects of rapamycin-induced autophagy on ITR resistance of *Candida albicans* under different states

**Figure 3:**
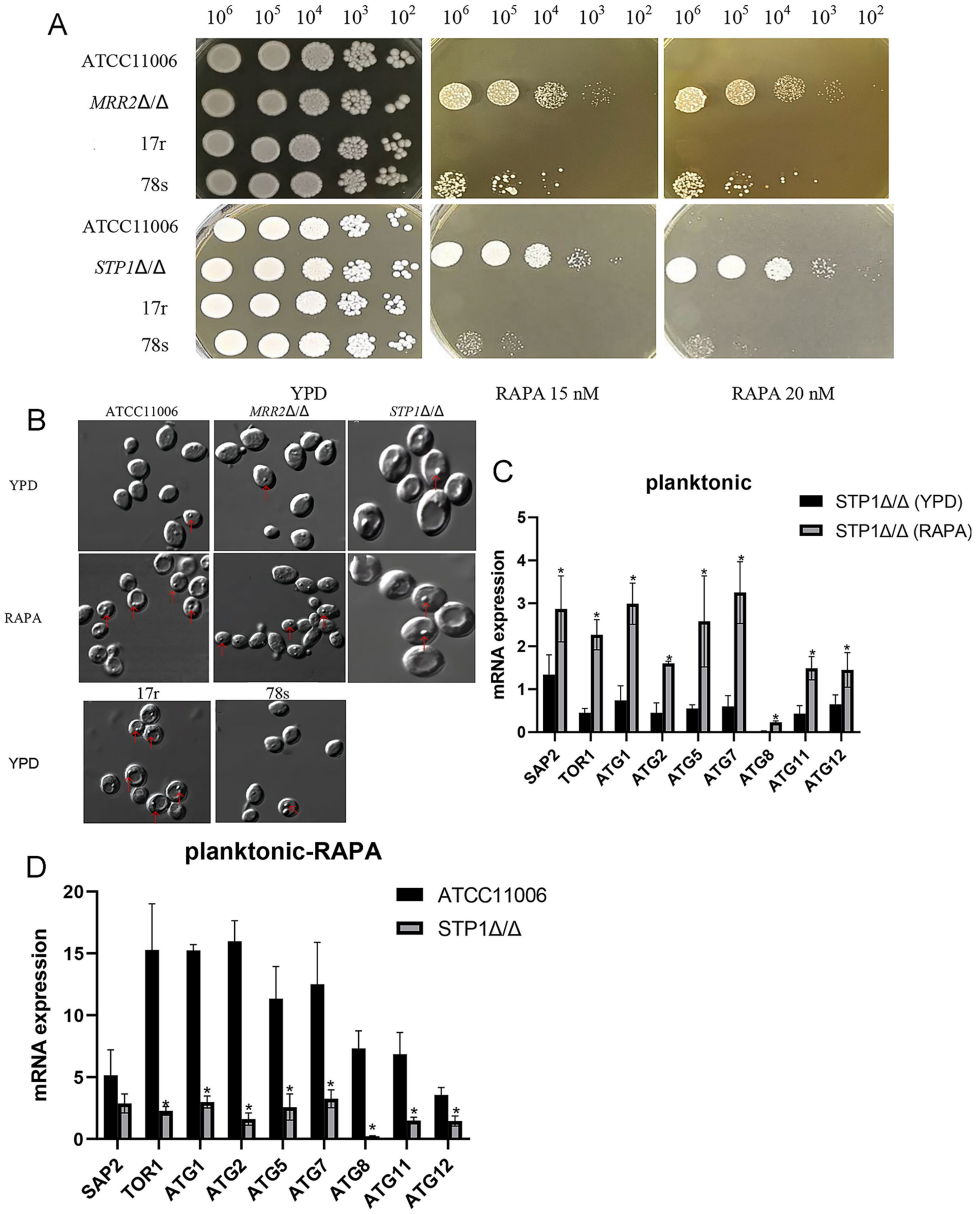
The effect of rapamycin-induced autophagy in *Candida albicans* on itraconazole resistance. **(A)** Growth trend of *Candida albicans* in YPD medium with different concentrations of rapamycin using spot assay. Cell concentrations were adjusted to 10^6^ cells/mL and were serially diluted at a ratio of 1:10, and 10 μL of each dilution was spotted onto agar plates with different concentrations of rapamycin (15 nM and 20 nM). **(B)** Formation of autophagosomes in *Candida albicans* ATCC11006, *MRR2*Δ/Δ, and *STP*1Δ/Δ strains before and after induction by rapamycin, as well as in drug-resistant and drug-sensitive strains under normal culture conditions (YPD medium, without rapamycin treatment) using a laser scanning confocal microscopy at the differential interference contrast (DIC) mode. Red arrows indicated autophagosomes. 78 s: a representative sensitive strain, 17r: a representative resistant strain. The mRNA expression of *SAP2* and autophagy-related genes in *STP*1Δ/Δ strains before **(C)** and after **(D)** rapamycin induction under planktonic conditions. * *p* < 0.05.

**Figure 4:**
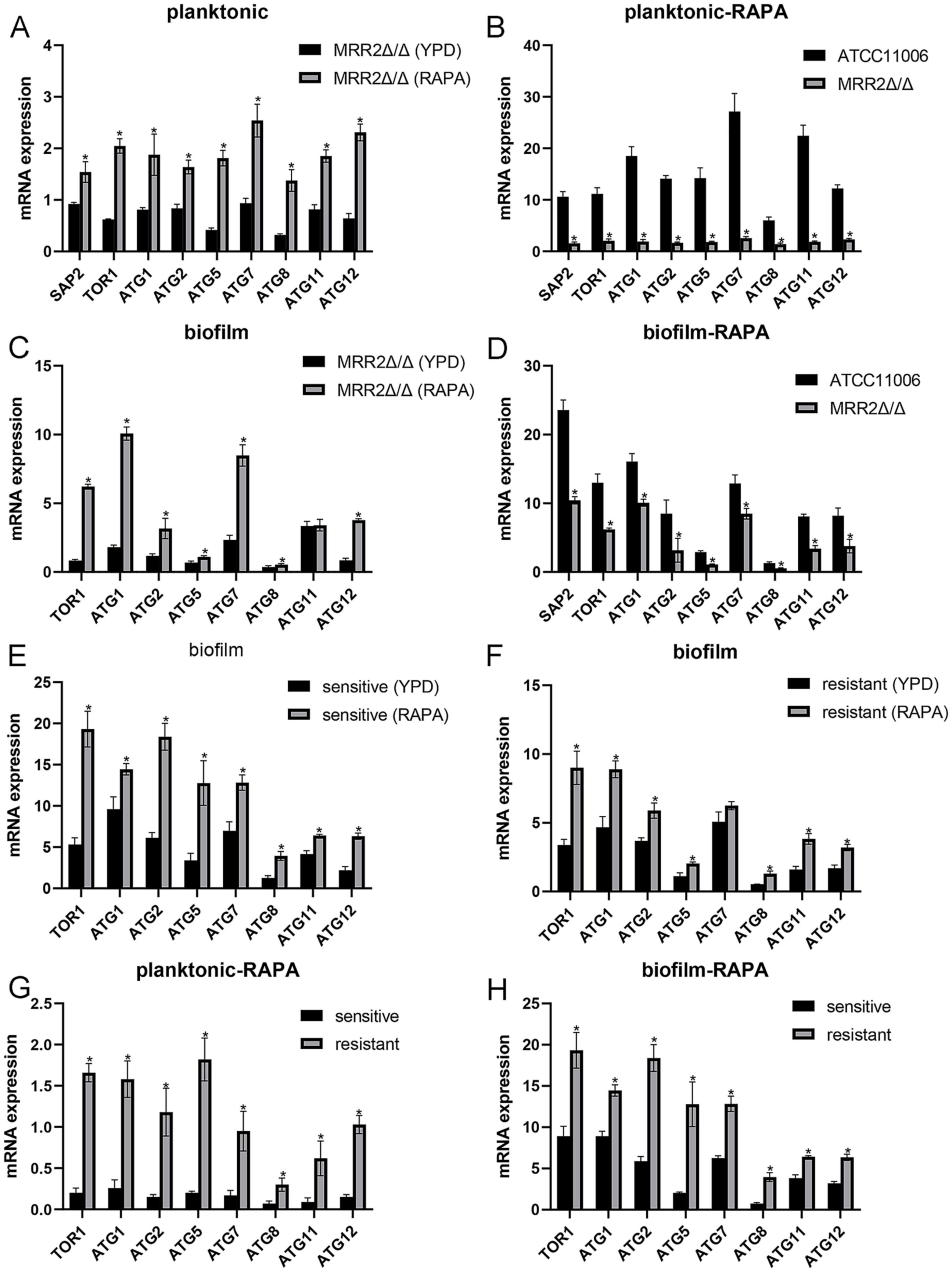
Comparison of *SAP2* and autophagy-related genes in different *Candida albicans* strains after rapamycin induction under different states. The mRNA expression of *SAP2* and autophagy-related genes in *MRR2*Δ/Δ strains before **(A)** and after **(B)** rapamycin induction under planktonic conditions. The mRNA expression of *SAP2* and autophagy-related genes in *MRR2*Δ/Δ strains before **(C)** and after **(D)** rapamycin induction under biofilm conditions. The mRNA expression of autophagy-related genes in sensitive **(E)** and resistant **(F)** strains before and after rapamycin induction under biofilm conditions. The mRNA expression of autophagy-related genes in sensitive and resistant strains after rapamycin induction under planktonic **(G)** and biofilm **(H)** conditions. * *p* < 0.05.

Rapamycin (RAPA), a classic autophagy inducer, was used to explore the role of autophagy in the regulation of *SAP2* expression and ITR resistance in *C. albicans* (Figure 3), and the expression profiles of *SAP2* and autophagy-related genes after rapamycin induction were further verified in different strains (Figure 4). The spot assay showed in the standard YPD medium, all experimental strains grew well with no significant differences in growth performance (Figure 3A). In contrast, distinct growth patterns were observed in YPD media supplemented with rapamycin at final concentrations of 15 nM and 20 nM, when compared with the standard YPD medium. The *STP1Δ/Δ* and *MRR2Δ/Δ* strains maintained normal growth in RAPA-containing YPD media, while the ITR-sensitive strains exhibited mild growth impairment, and the remaining strains showed significant growth defects (Figure 3A). These results indicated that the *STP1Δ/Δ* and *MRR2Δ/Δ* strains displayed a high tolerance to rapamycin, the ITR-sensitive strains had a moderate rapamycin tolerance, and the sensitivity of the strains to rapamycin was slightly enhanced with the increase in drug concentration. Under a microscopy at DIC mode, following induction with rapamycin at different concentrations, autophagosome formation was observed in the *STP1Δ/Δ* strain, *MRR2Δ/Δ* strain, ATCC11006 strain, as well as ITR-resistant (such as 17r) and ITR-sensitive (such as 78 s) strains (Figure 3B). However, the majority of autophagosomes in the standard ATCC11006 strain were successfully transported into and fused with the vacuoles, whereas autophagosomes in the *STP1Δ/Δ* and *MRR2Δ/Δ* strains failed to traffic to the vacuoles efficiently (Figure 3B). In addition, the *STP1Δ/Δ* and *MRR2Δ/Δ* strains exhibited a greater number of surface invaginations than the standard strain after rapamycin induction. Although intact autophagosomes were detected in both ITR-resistant and ITR-sensitive strains under YPD medium without rapamycin treatment, the number of autophagosomes was higher in ITR-resistant strains compared with ITR-sensitive strains (Figure 3B).

Then, the expression of autophagy-related genes was determined in different *C. albicans* strains after rapamycin induction under different states. At the planktonic state, the *STP1Δ/Δ* strain showed significant increased expression of *SAP2* and autophagy-related genes (*ATG1*, *ATG2*, *ATG5*, *ATG7*, *ATG8*, *ATG11*, *ATG12*) after rapamycin induction compared with those cultured in the standard YPD medium (*p* < 0.05, Figure 3C). In the strains after rapamycin induction under the planktonic condition, the expression of *SAP2* and all detected autophagy-related genes was evidently downregulated in the *STP1Δ/Δ* strains relative to the standard ATCC11006 (*p* < 0.05, Figure 3D).

For the *MRR2Δ/Δ* strain at the planktonic state, the expression of *SAP2* and all detected autophagy-related genes were significantly upregulated after rapamycin induction compared with those in strains cultured in YPD medium (Figure 4A); whereas in the strains induced by rapamycin, the expression of *SAP2* and all detected autophagy-related genes were evidently downregulated in the *MRR2Δ/Δ* strain in comparison with the ATCC11006 strain (*p* < 0.05, Figure 4B). At the biofilm state, rapamycin induction in the *MRR2Δ/Δ* strain significantly upregulated *SAP2* and most autophagy-related gene expression (*p* < 0.05, Figure 4C); while in the strains with rapamycin induction, their expression levels were markedly lower in the *MRR2Δ/Δ* strain than in the ATCC11006 strain (*p* < 0.05, Figure 4D).

For clinical strains in the biofilm state, rapamycin induction significantly upregulated the expression of autophagy-related genes in both ITR-sensitive (Figure 4E) and resistant (Figure 4F) strains relative to the strains cultured in YPD medium (*p* < 0.05). Comparative analysis after rapamycin induction showed that, under both planktonic (Figure 4G) and biofilm (Figure 4H) states, the expression of autophagy-related genes in resistant strains was significantly higher than that in sensitive strains (*p* < 0.05).

### 3.5 Effects of nitrogen starvation on the growth of *Candida albicans*

**Figure 5:**
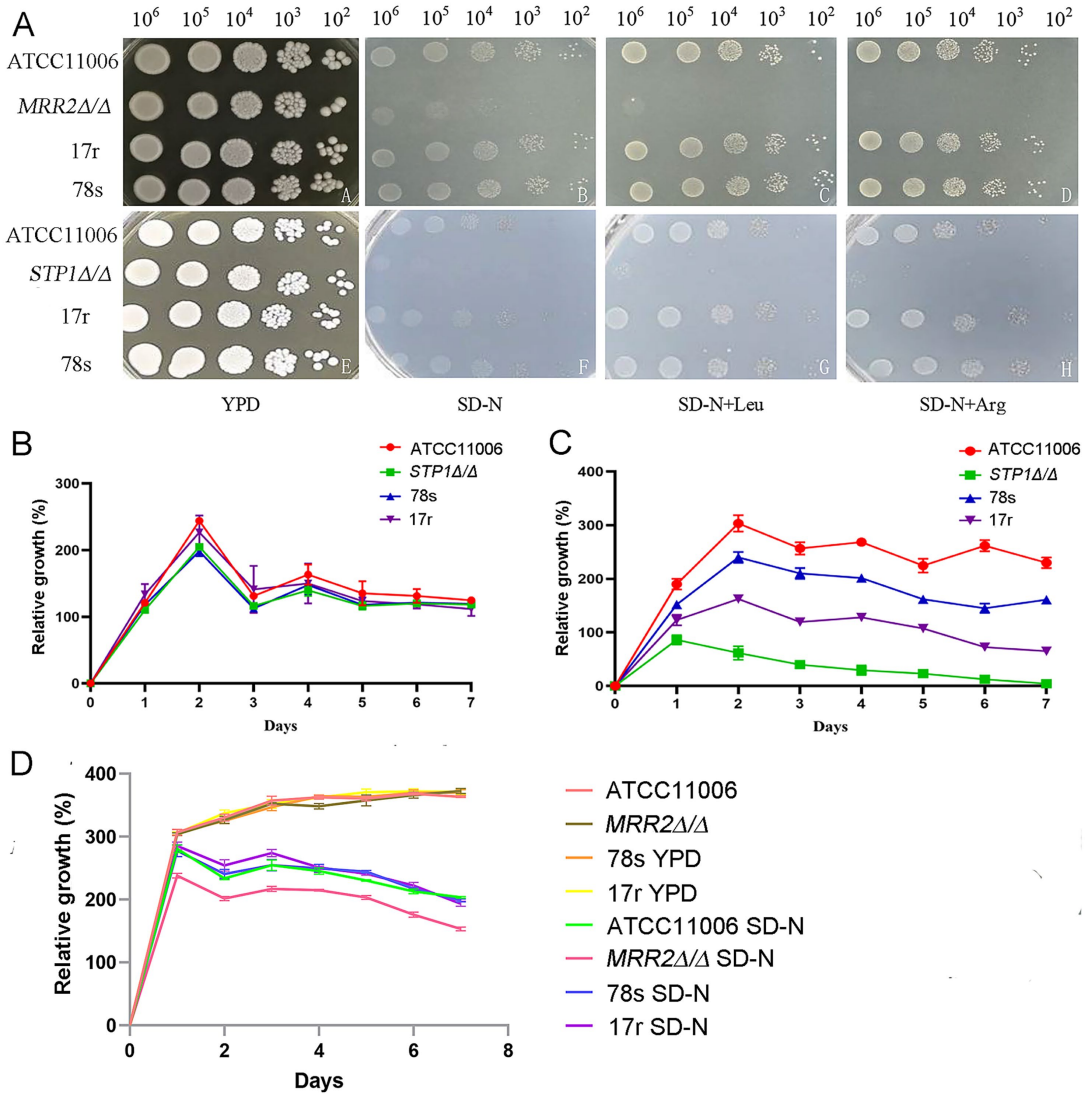
Effects of nitrogen starvation on the growth of *Candida albicans*. **(A)** Growth trends of *Candida albicans* (ATCC11006, *STP*1Δ/Δ, *MRR2*Δ/Δ), representative sensitive strain 78 s and representative resistant strain 17r in YPD medium, SD-N medium, and SD-N medium, respectively, supplemented with Leu and Arg using spot assay. Cell concentrations were adjusted to 10^6^ cells/mL and were serially diluted at a ratio of 1:10. **(B)** Relative growth curves of ATCC11006, *STP*1Δ/Δ strain, representative sensitive strain 78 s and representative resistant strain 17r in YPD medium. **(C)** Relative growth curves of ATCC11006, *STP*1Δ/Δ strain, representative sensitive strain 78 s and representative resistant strain 17r under nitrogen starvation over time. **(D)** Fungal growth curves of ATCC11006, *MRR2*Δ/Δ strain, representative sensitive strain 78 s and representative resistant strain 17r in YPD medium, and SD-N medium. 78 s: a representative sensitive strain, 17r: a representative resistant strain.

Given that *STP1* and *MRR2* are key regulators of nitrogen metabolism in *C. albicans*, spot assay and fungal growth curve analysis were used to detect the growth tolerance of different strains under nitrogen starvation (SD-N medium), and the rescue effect of amino acid supplementation (Leu, Arg) was also explored (Figure 5). Strain 78 s was defined as the representative ITR-sensitive strain, and strain 17r as the representative ITR-resistant strain.

The spot assay showed the growth of the *STP1Δ/Δ* strain, *MRR2Δ/Δ* strain, sensitive strain 78 s and resistant strain 17r in YPD, SD-N, SD-N + Leu and SD-N + Arg media (Figure 5A). All strains grew well in YPD medium; as well as under nitrogen starvation (SD-N), the growth of the *STP1Δ/Δ* and *MRR2Δ/Δ* strains were significantly inhibited, while the growth trends of other strains are roughly the same. The growth trend of SD-N medium with added Leu and medium with added Arg hydrochloride was observed to be the same as that of SD-N medium. Quantitative analysis further confirmed that YPD medium had no significant effect on the growth of these three strains (Figure 5B), while under nitrogen starvation cultivation conditions, ATCC11006, sensitive (such as 78 s) and resistant (such as 17r) strains could grow normally, but the growth of *STP1*∆/∆ strains gradually weakened with prolonged cultivation time (Figure 5C), indicating that the deletion of *STP1* gene could increase the sensitivity of strains to nitrogen starvation. Compared with the relative growth rate of sensitive strains, the growth trend of resistant strains decreased, indicating that nitrogen starvation may be one of the reasons for fungal resistance. In addition, the *MRR2*Δ/Δ strain had a significantly lower relative growth rate in SD-N medium than in YPD medium over time, and the relative growth rate decreased rapidly with the extension of nitrogen starvation time (Figure 5D), indicating that MRR2 knockout significantly reduced the nitrogen starvation tolerance of *C. albicans*.

## 4 Discussion

*C. albicans* is the most prevalent opportunistic fungal pathogen in clinical settings, and its biofilm formation and azole antifungal resistance have become core challenges for clinical anti-candidiasis therapy (40, 41). Biofilm formation endows *C. albicans* with strong resistance to host immunity and antifungal drugs, while autophagy, as a conserved intracellular catabolic process, is increasingly recognized as a key regulatory node linking fungal stress response, biofilm development and drug resistance (24, 42). In this study, we systematically explored the roles of *STP1* and *MRR2* in *C. albicans* biofilm formation and ITR resistance, and clarified their regulatory mechanisms through *SAP2* expression and autophagic activity. Our findings provide new molecular targets for the development of anti-candidiasis strategies targeting biofilm and drug resistance.

Biofilm formation is a complex developmental process of *C. albicans* involving adhesion, yeast cell proliferation, hyphal differentiation, extracellular matrix secretion and mature biofilm construction (13). Abnormal regulation of this process is closely related to the enhanced pathogenicity and drug resistance of clinical isolates. Our study found that the biofilm-forming ability of the *STP1Δ/Δ* strain was significantly enhanced, while that of the *MRR2Δ/Δ* strain was significantly reduced compared with the standard ATCC11006 strain. Morphological observation further showed that both knockout strains exhibited higher hyphal aggregation at the mature biofilm stage (48 h), and hyphae of ITR-resistant clinical strains were more densely packed than those of sensitive strains. This result is consistent with previous studies indicating that hyphal formation and aggregation are the structural basis of *C. albicans* biofilm maturation, and enhanced hyphal development is positively correlated with the biofilm-forming ability and drug resistance of strains (43, 44). Notably, local hyphal aggregation observed under confocal microscopy represents morphological characteristics in representative fields, which is not equivalent to total biofilm biomass determined by crystal violet staining. The *MRR2*Δ/Δ strain showed enhanced local hyphal aggregation but reduced overall adhesion and mature biofilm formation, resulting in lower OD490 values. In contrast, the *STP1*Δ/Δ strain displayed both increased hyphal aggregation and higher total biofilm biomass. These distinct phenotypes indicate that *STP1* and *MRR2* regulate biofilm formation through different mechanisms affecting hyphal organization, structural stability, and total biomass separately.

For *STP1*, as a zinc finger family transcription factor regulating nitrogen metabolism and the SPS amino acid sensing pathway (45), its knockout-induced enhancement of biofilm formation may be related to compensatory activation of fungal stress response pathways. Previous studies have shown that deletion of nitrogen metabolism-related genes can induce upregulation of fungal virulence factor expression and enhance biofilm formation (46, 47), which is consistent with our observation that STP1 knockout promotes biofilm formation. For *MRR2*, as a putative magnesium transporter gene, a previous study confirmed its role in fluconazole resistance by regulating the efflux pump gene CDR1 (48), and this study further identified its positive regulatory effect on biofilm formation. The reduced biofilm-forming ability of the *MRR2Δ/Δ* strain may be related to impaired magnesium ion transport, which affects cell wall synthesis and hyphal formation in *C. albicans*. Magnesium serves as an important cofactor for cell wall synthetases, disturbed transport leads to abnormal fungal cell wall structure and inhibited hyphal development (49, 50), thereby reducing the biofilm-forming ability. In addition, the significantly enhanced biofilm formation ability of ITR-resistant clinical strains further verified the clinical correlation between biofilm formation and azole resistance, which is consistent with the clinical observation that biofilm-forming *C. albicans* isolates have higher drug resistance and mortality (51). These results suggested that *STP1* and *MRR2* could be key regulators of *C. albicans* biofilm formation with opposite regulatory effects.

SAP2, a key member of the Sap family, is a core virulence factor of *C. albicans* involved in biofilm formation, host tissue invasion and antifungal resistance (52). Our study found that the expression levels of *SAP2*, *STP1* and *MRR2* in ITR-resistant strains were significantly higher than those in sensitive strains under both planktonic and biofilm states, and the expression of all three genes was significantly upregulated in the biofilm state compared with the planktonic state. These results indicated that high expression of *SAP2*, *STP1* and *MRR2* may be closely related to the ITR resistance and biofilm formation in *C. albicans*, which is consistent with a previous research that *SAP2* overexpression can enhance the invasive ability and drug resistance of *C. albicans* by reducing the adhesion of early biofilm and promoting the degradation of host extracellular matrix (53). Correlation analysis further revealed the growth state-dependent regulatory relationship between *STP1*/*MRR2* and *SAP2*. This phenomenon suggested that the regulation of *SAP2* by *STP1* and *MRR2* may be a complex process affected by the fungal growth state. Under planktonic conditions, *MRR2* may directly regulate the transcription of *SAP2* or indirectly promote its expression by regulating the efflux pump pathway (54), thus showing a significant positive correlation. In contrast, under biofilm state, the formation of complex microbial communities and the secretion of extracellular matrix lead to the activation of multiple signaling pathways in fungi (55). The regulation of *SAP2* is transformed into the synergistic effect of multiple transcription factors (such as CAP1, GCN4 (31, 32)), resulting in the loss of a simple linear correlation between *STP1/MRR2* and *SAP2*. The marginally positive correlation between *SAP2* and *STP1* under biofilm conditions (*p* = 0.054) suggested that *STP1* may still participate in the regulation of *SAP2* in the biofilm state, but its regulatory effect is weakened by other pathways, which requires further verification by chromatin immunoprecipitation and other assays. Taken together, we speculate that *STP1/MRR2* may regulate the expression of virulence factor *SAP2*, and their correlations may be dependent on growth state.

Autophagy is an important stress response mechanism in *C. albicans*, which can promote fungal growth under nutrient starvation, drug stress and other adverse conditions, and is closely related to biofilm formation and drug resistance (56, 57). Our study found that the expression of key autophagy-related genes (*ATG1*, *ATG2*, *ATG5*, *ATG7*, *ATG8*, *ATG11*, *ATG12*, *TOR1*) in *STP1Δ/Δ* and *MRR2Δ/Δ* strains was significantly lower than that in the ATCC11006 strain under both planktonic and biofilm states, while the expression of these genes in ITR-resistant strains was significantly higher than that in sensitive strains. Meanwhile, the autophagy-related gene expression was significantly higher in the biofilm state than that in the planktonic state in all tested strains. These results indicated that *STP1* and *MRR2* are associated with the expression of autophagy-related genes in *C. albicans*, and their expression levels may be positively correlated with the biofilm formation ability and ITR resistance of strains.

The observed association between *STP1* and autophagy-related gene expression is consistent with its role in nitrogen metabolism and the TOR signaling pathway, which is a known upstream regulator of autophagy. *STP1* is a key gene of the TOR signaling pathway upstream of autophagy (58), and the activation of TOR pathway can inhibit the occurrence of autophagy (59). Deletion of *STP*1 may lead to the abnormal activation of TOR pathway, thus inhibiting the expression of autophagy-related genes. However, direct evidence supporting this inference is current lacking. For *MRR2*, the reduced expression of autophagy-related genes in the deletion mutant may be related to impaired magnesium ion transport: magnesium is crucial fungal intracellular ATP synthesis and signal transduction (60), and the transport disorder caused by *MRR2* knockout will lead to the inhibition of autophagic activity by reducing the energy supply of autophagy process. These mechanistic hypotheses require further experimental verification. In addition, the higher autophagic activity of ITR-resistant strains and biofilm state strains further confirms the research conclusion that autophagy promotes the drug resistance of *C. albicans*. Autophagy can remove the damaged organelles and proteins caused by antifungal drugs, promote the repair of fungal cells, and at the same time provide nutrients for the growth and metabolism of fungi in biofilm, thus enhancing the drug resistance and growth ability of strains (22, 61). These reports, combined with our outcomes, it can be inferred that *STP1* and *MRR2* may be associated with the expression of autophagy-related genes in *C. albicans*, and autophagy may be positively correlated with ITR resistance.

Rapamycin as a classic autophagy inducer, can specifically inhibit the TOR pathway and activate autophagy in *C. albicans* (62), which is an important tool to study the relationship between autophagy and fungal drug resistance. Our study found that *STP1Δ/Δ* and *MRR2Δ/Δ* strains had high tolerance to rapamycin, while ITR-sensitive strains had mild growth impairment. DIC microscopy observation further found that rapamycin induction could promote the formation of autophagosomes in all tested strains; however, the autophagosomes of *STP1Δ/Δ* and *MRR2Δ/Δ* strains failed to effectively fuse with vacuoles, and the number of surface invaginations was higher than that of the standard strain. In addition, molecular expression analysis showed that rapamycin induction could significantly upregulate the expression of *SAP2* and autophagy-related genes in *STP1Δ/Δ* and *MRR2Δ/Δ* strains under planktonic and biofilm states, but the expression level was still significantly lower than that in ATCC11006 strain. Meanwhile, rapamycin induction significantly upregulated the autophagic gene expression of ITR-resistant strains, and the expression level was significantly higher than that of sensitive strains. These results suggest that STP1 and MRR2 may regulate SAP2 expression by affecting the transcription of autophagy-related genes, thereby indirectly influencing biofilm formation and ITR resistance (46). Under drug stress, the high expression of *STP1* and *MRR2* may activate autophagy, promote the high expression of *SAP2,* enhance the biofilm formation ability of fungi, and thus improve the ITR resistance. In addition, the upregulation of *SAP2* expression by rapamycin-induced autophagy also suggests that autophagy may be an important upstream regulatory pathway of *SAP2*, which enrich the molecular regulatory network of *SAP2*.

Nitrogen starvation is an important environmental stress encountered by *C. albicans* faces in the host niches, and adaptation to nitrogen starvation is closely related to the pathogenicity and drug resistance of fungi (63). Our study found that the growth of *STP1Δ/Δ* and *MRR2Δ/Δ* strains was significantly inhibited under nitrogen starvation (SD-N) medium, and the relative growth rate of *STP1Δ/Δ* and *MRR2Δ/Δ* strain decreased rapidly with the extension of starvation time. As a key regulator of nitrogen metabolism, *STP1* deletion directly leads to the disorder of fungal nitrogen utilization (64), while *MRR2* deletion affects the nitrogen metabolism process by interfering with magnesium transport, thus reducing the tolerance to nitrogen starvation. In addition, ITR-resistant strains showed better growth tolerance under nitrogen starvation than sensitive strains, indicating that enhanced nitrogen starvation adaptability contributes to the high drug resistance and pathogenicity of resistant strains. Fungi can maintain their growth and biofilm formation under nutrient-limited conditions in the host by enhancing the nitrogen starvation tolerance, thus evading the killing of antifungal drugs (65). It is worth noting that the supplementation of L-arginine and L-leucine did not effectively rescue the growth of *STP1Δ/Δ* and *MRR2Δ/Δ* strains under nitrogen starvation, possibly because *STP1* and *MRR2* deletion leads to the impairment of the fungal amino acid transport and utilization pathways (33, 48), so the exogenous amino acid supplementation cannot compensate for the nitrogen metabolism disorder caused by gene knockout. These results further suggest that the nitrogen metabolism pathway regulated by STP1 and MRR2 may be a key target for regulating fungal stress response and drug resistance of *C. albicans*.

However, there are some limitations in our study. First, this study only verified the regulatory relationship between *STP1/MRR2* and *SAP2* at the transcriptional level, and the direct binding of *STP1* and *MRR2* to the *SAP2* promoter region needs to be further confirmed by ChIP and dual-luciferase reporter gene assays. Second, our results only show that *STP1* and *MRR2* deletion is associated with decreased transcription of autophagy-related genes, but direct evidence is lacking to confirm that *STP1* and *MRR2* negatively regulate autophagic activity. Third, we did not provide direct experimental evidence to verify abnormal activation of the TOR pathway caused by *STP1* deletion, or energy deficiency caused by *MRR2* deletion. These mechanistic hypotheses need to be further verified by autophagic flux detection, protein phosphorylation analysis, ATP level detection, and gene complementation assays. Fourth, this study is an *in vitro* experimental study, and the regulatory roles of *STP1* and *MRR2* in the pathogenicity and drug resistance of *C. albicans* need to be further verified by *in vivo* animal models. Additionally, the downstream signaling pathways of *STP1* and *MRR2* in regulating autophagy and *SAP2* expression need to be further explored, and the interaction between *STP1* and *MRR2* is also worthy of in-depth study.

In conclusion, our study confirms that *STP1* and *MRR2* may be key regulatory genes of *C. albicans* biofilm formation and ITR resistance. These two genes were associated with the expression of the virulence factor *SAP2* and the transcriptional regulation of autophagy-related genes, thereby affecting biofilm development and antifungal resistance. Our findings support that *STP1* and *MRR2* are likely involved in the transcriptional regulation of autophagy-related genes, but direct evidence is still lacking to confirm that they negatively regulate autophagic activity. Autophagic activity may be positively correlated with the biofilm formation ability and ITR resistance of *C. albicans*, and rapamycin-induced autophagy can upregulate *SAP2* expression and enhance drug resistance. In addition, *STP1* and *MRR2* are essential for *C. albicans* to tolerate nitrogen starvation, and the nitrogen metabolism pathway regulated by the two genes may be closely linked to fungal drug resistance. This study enriches the molecular regulatory network of *C. albicans* biofilm formation and azole resistance, and provides a new theoretical basis and molecular target for the development of anti-candidiasis strategies targeting autophagy and nitrogen metabolism pathways.

## Data Availability

The original contributions presented in the study are included in the article/supplementary material, further inquiries can be directed to the corresponding author/s.
